# The Protective Effects of Influenza Vaccination in Elderly Patients with Breast Cancer in Taiwan: A Real-World Evidence-Based Study

**DOI:** 10.3390/vaccines10071144

**Published:** 2022-07-19

**Authors:** Szu-Yuan Wu, Ho-Jui Tung, Kuang-Hua Huang, Chiachi Bonnie Lee, Tung-Han Tsai, Yu-Chia Chang

**Affiliations:** 1 Department of Healthcare Administration, College of Medical and Health Science, Asia University, Taichung 41354, Taiwan; szuyuanwu5399@gmail.com; 2 Department of Food Nutrition and Health Biotechnology, College of Medical and Health Science, Asia University, Taichung 41354, Taiwan; 3 Big Data Center, Lo-Hsu Medical Foundation, Lotung Poh-Ai Hospital, Yilan 265501, Taiwan; 4 Division of Radiation Oncology, Lo-Hsu Medical Foundation, Lotung Poh-Ai Hospital, Yilan 265501, Taiwan; 5 Graduate Institute of Business Administration, College of Management, Fu Jen Catholic University, Taipei 242062, Taiwan; 6 Department of Management, College of Management, Fo Guang University, Yilan 262307, Taiwan; 7 Centers for Regional Anesthesia and Pain Medicine, Wan Fang Hospital, Taipei Medical University, Taipei 11696, Taiwan; 8 Department of Health Policy and Community Health, Jiann-Ping Hsu College of Public Health, Georgia Southern University, Statesboro, GA 30460, USA; htung@georgiasouthern.edu; 9 Department of Health Services Administration, China Medical University, Taichung 40402, Taiwan; khhuang@mail.cmu.edu.tw (K.-H.H.); bonnielee@mail.cmu.edu.tw (C.B.L.); dondon0525@gmail.com (T.-H.T.); 10 Department of Long Term Care, College of Health and Nursing, National Quemoy University, Kinmen 892009, Taiwan

**Keywords:** influenza vaccination, elderly, breast cancer, mortality, hospitalization

## Abstract

In elderly patients with newly diagnosed breast cancer, clarity is lacking regarding the effects of influenza vaccines, particularly on clinical outcomes. This study conducted two nationwide, population-based, and propensity score-matched cohorts to estimate and compare the protective effects of influenza vaccine in elderly women and elderly patients with breast cancer. Data were derived from the National Health Insurance Research Database and Cancer Registry Database. Generalized estimating equations (GEEs) were used to compare outcomes between the vaccinated and unvaccinated cohorts. Adjusted odds ratios (aORs) were used to estimate the relative risks, and stratified analyses in the breast cancer cohort were performed to further evaluate elderly breast cancer patients undergoing a variety of adjuvant therapies. The GEE analysis showed that the aORs of death and hospitalization, including for influenza and pneumonia, respiratory diseases, respiratory failure, and heart disease, did not significantly decrease in vaccinated elderly patients with newly diagnosed breast cancer. Conversely, the aORs of all influenza-related clinical outcomes were significantly decreased in elderly women. No protective effects of influenza vaccination were found in the elderly patients with a newly diagnosed breast cancer. More studies focusing on identifying strategies to improve the real-world effectiveness of influenza vaccination to the immunocompromised are needed. Our clinical outcomes will be valuable for future public health policy establishment and shared decision making for influenza vaccine use in elderly patients with newly diagnosed breast cancer. According to our findings, regular influenza vaccine administration for elderly patients with newly diagnosed breast cancer may be reconsidered, with potential contraindications for vaccination. On the other hand, implementing the vaccination of close contacts of patients with breast cancer may be a more important strategy for enhancing protection of those fragile patients.

## 1. Introduction

Infection prevention is paramount to the ever-increasing population of patients with cancer who have impaired immunity [1]. In these patients, infection often results in excessive morbidity and mortality, and antimicrobial therapy is often less effective in these patients than in those with intact immunity [1]. Although immunization appears to be an obvious method of infection prevention, many cancer patients with impaired immunity do not exhibit a protective immune response to active vaccination [2,3]. Furthermore, immunization with live-virus vaccines may result in unchecked proliferation of attenuated strains.

Patients with cancer who are receiving immunosuppressive therapy should not be administered live virus vaccines [2,3]. However, physicians favor the administration of an inactivated influenza vaccine to patients with cancer given the need for protection against circulating seasonal strains of influenza [2,4,5]. However, other physicians do not recommend it because the immune response to the influenza vaccine is likely to be impaired in patients with cancer receiving systemic treatments [2,3]. These recommendations are based on limited data from patients with different solid tumors receiving chemotherapy, suggesting that immunization on day 4 or 5 of a chemotherapy cycle is more immunogenic than on day 16 [6,7].

Breast cancer is the most common cancer in women and the leading cause of cancer-related death among women in Taiwan [8]. The major treatment protocol is the surgical removal of breast cancer and suspected lymph nodes [9]. Adjuvant therapy includes chemotherapy, hormone therapy, radiotherapy, or target therapy, depending on the molecular expression of the hormone receptor, human epidermal growth factor receptor (HER2) receptor, and clinical stage [10,11,12].

The first-line treatments for early-stage breast cancer are relatively consistent, and the life expectancy of patients treated for breast cancer is longer than that of those treated for other cancers [8,10,11,12,13]. Therefore, the prevention of mortality, influenza-related emergency admission, hospitalization, or inpatient expenditure in patients with breast cancer is crucial. In addition, medical care consumption in breast cancer treatment is high in Taiwan [14]. Decreasing the medical expenditures associated with influenza-associated complications in patients with breast cancer would be valuable for health policy establishment and government expenditure reduction.

The results regarding the immunogenicity of immunization in patients with cancer have been inconsistent, with one study showing poor immunogenicity [3] but another study showing good immunogenicity [15]. All influenza vaccine studies in patients with cancers have been based on the antibody response after influenza vaccination, instead of clinical outcomes, such as influenza-related complications or death [2,3,6,7,16,17,18,19,20,21,22]. Previous influenza vaccine studies have included various cancer types and different cancer treatments [2,3,6,7,16,17,18,19,20,21,22].

Different treatments in various cancers lead to different immunocompromised conditions. Moreover, all the aforementioned studies have been associated with a small sample size of patients with cancer, heterogeneous cancer types, and short-term follow-up [2,3,6,7,16,17,18,19,20,21,22]. Therefore, we used head-to-head propensity score-matched (PSM) cohorts to mimic a randomized controlled trial (RCT) [23] for examining the real-world protective effects of influenza vaccination in the prevention of influenza-related complications and reducing the related expenditure in elderly patients with newly diagnosed breast cancer. Furthermore, in order to make the research results more reliable, we included a second cohort that consisted of general elderly women aged 65 and over who had no cancer diagnosis as a positive control cohort.

## 2. Materials and Methods

### 2.1. Background Information

In Taiwan, a universal insurance scheme called the National Health Insurance (NHI) was launched in 1995, which is a single-payer national health insurance plan that covers >99.9% of the citizens of Taiwan [24]. Free influenza vaccination for elderly people aged ≥65 years has been implemented through the NHI program since 2001 [25,26]. Each year, starting from October 1, enrolled elderly people can visit any NHI-licensed clinic or hospital to receive free influenza vaccination [25,26,27] based on the Taiwan’s Annual Seasonal Influenza Mass Vaccination Program-Lessons for Pandemic Planning [27].

### 2.2. Study Design

This study was a population-based retrospective cohort study. The data used in the analysis were obtained from the National Health Insurance Research Database (NHIRD) released by the Health and Welfare Data Science Center, Ministry of Health and Welfare Taiwan. The NHIRD includes detailed clinical records on outpatient visits, hospitalizations, diagnostic codes, and prescriptions [28]. Data are deidentified before releasing it to researchers; consequently, individual privacy is protected. Our protocols were reviewed and approved by the Institutional Review Board (IRB) of the Taichung Jen-Ai Hospital, Taiwan (Approval date: 8 January 2019, No.107-49).

### 2.3. Two Population-Based Study Cohorts

To examine the real-world protective effect of influenza vaccination, we enrolled two population-based elderly cohorts (aged 65 years and older) for comparison. One cohort consisted of elderly patients with newly diagnosed breast cancer and the other consisted of general elderly women (excluded patients with cancer diagnosis). Participants in both cohorts included elderly women from the NHIRD between 1 January 2010, and 31 December 2016.

The cohort of elderly women with breast cancer was selected using the NHIRD linked to the Taiwan Cancer Registry Database (TCRD). All elderly patients with breast cancer had received curative surgery for the removal of breast cancer and suspected lymph nodes [12,29,30] before the free influenza vaccination period (between 1 October and 31 December).

We excluded men, those <65 years old, those who received influenza vaccination between January 1 and September 30 (outside the free influenza vaccination period), those with other cancer before the breast cancer diagnosis date, those who received influenza vaccination two times or more within 1 year, and those who died during the influenza vaccination period. Furthermore, we excluded those with carcinoma in situ, metastasis, and other cancer diagnosis from 2010 to 2016, as well as these with unclear cancer stages from the elderly breast cancer cohort. We finally included 10,825 elderly patients with newly diagnosed breast cancer and categorized them into influenza vaccinated and unvaccinated groups.

To increase the efficiency of comparisons, PSM was used to reduce confounding and selection bias [23]. Regarding the PSM method, the multivariate logistic regression model, which included observed covariates (age, premium-based monthly salary, urbanization, Charlson Comorbidity Index [CCI] score, and clinical cancer stages) was applied to obtain propensity scores for the probabilities of being in the vaccinated and unvaccinated groups. The 1:2 matching process yielded a final cohort of 1191 elderly patients with newly diagnosed breast cancer who received influenza vaccination and 3982 who did not receive influenza vaccination. The flowchart of the selection of elderly patients with breast cancer is presented in Figure S1.

As for the elderly general women cohort, the enrollment criteria were similar to cohort of elderly patients with breast cancer. After PSM method with a 1:1 matching process yielded a final cohort of 585,327 participants each in the vaccinated and unvaccinated cohorts. The flowchart of the selection of the elderly general population cohort is presented in Figure S2.

### 2.4. Independent Variable

The independent variable, that is, whether participants received the influenza vaccine or not, was determined based on the NHIRD claims data (confirmed using drug codes) between 1 October and 31 December from 2010 to 2016, when seasonal influenza vaccines were freely available every year to people aged ≥65 years in Taiwan. This variable was dichotomized and coded as 1 (received influenza vaccine) and 0 (did not receive influenza vaccine).

### 2.5. Outcome Measures

Four outcome indicators were used to evaluate the effectiveness of the seasonal influenza vaccine, namely all-cause mortality, emergency admission, hospitalization, and inpatient expenditure due to influenza-related complications during the influenza season. All-cause mortality was determined from the Cause of Death Registry.

Claim records from the NHIRD were used to determine emergency admission, hospitalization, and inpatient expenditures due to influenza-related complications, including influenza and pneumonia (International Classification of Diseases, Ninth Revision, Clinical Modification [ICD-9-CM] codes 480–487; International Classification of Diseases, Tenth Revision, Clinical Modification [ICD-10-CM] codes J09–J18), respiratory diseases (ICD-9-CM codes 460–466, 480–487, 490–496, and 500–518; ICD-10-CM codes J00–J06, J09–J18, J40–J45,J47, J60–J70, J80–J86, J90–J99), respiratory failure (ICD-9-CM codes 518.81–518.84, 799.1; ICD-10-CM codes J96.0–J96.2, J96.9, R09.2), and heart diseases (ICD-9-CM codes 410–429; ICD-10-CM codes I20–I52).

Inpatient expenditures for influenza-related complications were defined as the sum of inpatient medical expenses, including fees for physician and nurse care, surgeries or procedures, medications, examinations and tests, and hospital stay as well as copayments and other miscellaneous fees [31]. The influenza season was defined based on the influenza-like illness (ILI) activity peak of the influenza-surveillance data from the Centers for Disease Control (CDC) in Taiwan [25]. From 2010 to 2016, the CDC surveillance system indicated that ILI activities were not similar every year; however, in general, it began to increase at the beginning of January and plateaued at the end of September [25,26]. Therefore, the influenza season in this study was defined as the period between January and September every year from 2010 to 2016.

### 2.6. Covariates

Baseline covariates were examined for both the breast cancer and comparison cohorts, including age, premium-based monthly salary, urbanization, CCI score, and health care utilization in the past year (number of outpatient visits, hospitalization, and influenza vaccination status). Participants were divided into three groups according to age: 65–69, 70–74, and ≥75 years. Premium-based monthly salaries were divided into ≤20,008 New Taiwan Dollars (NTD); 20,009–22,800 NTD; 22,801–38,200 NTD; and ≥38,201 NTD (1 NTD is approximately equal to 0.33 USD).

Urbanization of the area of residence was stratified into seven levels: levels 1 to 7. Level 1 represented the highest degree of urbanization, and level 7 represented the least. Furthermore, variables related to cancer treatment in the breast cancer cohort included clinical stages of cancer, radiotherapy, chemotherapy, hormone treatment, and targeted therapy. The clinical stage was assigned according to the American Joint Committee on Cancer, seventh edition.

### 2.7. Statistical Analysis

Bivariable comparisons of the covariates and influenza status were performed using chi-square tests. The generalized estimating equation (GEE) was used to compare the four outcomes between the vaccinated and unvaccinated cohorts. GEE with binomial distribution was used to estimate the adjusted odds ratios (aORs) of receiving influenza vaccination for three clinical outcome measures (all-cause death, emergency admission, and hospitalization).

Stratified analyses were performed on selected adjuvant therapies (e.g., chemotherapy, radiotherapy, target therapy, or hormone therapy) to examine if these treatment procedures were associated with the effectiveness of influenza vaccination. In addition, because the inpatient expenditure appeared to be right-skewed, the GEE model with a log-link function and gamma distribution was incorporated. All analyses were performed using SAS version 9.4 (SAS Institute, Inc., Cary, NC, USA). A two-tailed *p* value of 0.05 was considered statistically significant.

## 3. Results

### 3.1. Descriptive Results

Table 1 presents a comparison of baseline characteristics between the vaccinated and unvaccinated groups of two cohorts. After application of the PSM method, no significant differences were found between the two groups. Table 2 and Table 3 present the results using the PSM sample. The most common clinical stages of breast cancer in our cohort were stages I–II in approximately 85% of participants.

### 3.2. The Results of Elderly Patients with Newly Diagnosed Breast Cancer

No statistically significant differences were observed in all-cause mortality, emergency admission, and hospitalization between influenza vaccinated and unvaccinated patients with breast cancer (Table 2).

Table 3 shows no significant differences in inpatient expenditure due to influenza and pneumonia, respiratory diseases, respiratory failure, and heart disease between influenza vaccinated and nonvaccinated patients with breast cancer. Inpatient expenditure for influenza and pneumonia, respiratory diseases, and respiratory failure was lower in breast cancer patients with influenza vaccination than in those without influenza vaccination, although the aORs of the GEE analysis did not reach statistical significance between influenza vaccinated and unvaccinated groups.

**Figure 1:**
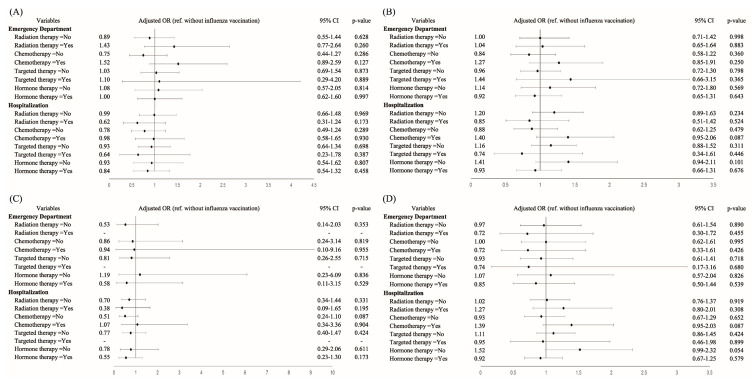
Subgroup analysis of protective effects of influenza vaccination in breast cancer patients with different adjuvant treatments, such as chemotherapy, radiotherapy, target therapy, or hormone therapy. ‘Yes’ means patients receiving this adjuvant treatment, and ‘No’ means patients not receiving this adjuvant treatment. Risk of (**A**) influenza and pneumonia, (**B**) respiratory diseases, (**C**) respiratory failure, and (**D**) heart disease in patients with breast cancer.

For vaccinated and unvaccinated elderly patients with breast cancer undergoing different adjuvant treatments, we used the forest plots of the aORs for influenza-related complications, stratified by chemotherapy, radiotherapy, target therapy (HER2 inhibitors), and hormone therapy. On the basis of the findings in Figure 1, no significant protective effect of the influenza vaccine was observed in elderly patients with newly diagnosed breast cancer receiving influenza vaccination in the year of breast cancer diagnosis, irrespective of the breast-cancer-treatment type.

### 3.3. Results of General Elderly Women Cohort

Adjusted GEE analyses revealed that the aOR of all-cause mortality was significantly lower for the vaccinated group than for the unvaccinated group (aOR = 0.58; 95% confidence interval [CI], 0.56–0.59). General elderly women in the vaccinated group had significantly lower risks of emergency admission for influenza and pneumonia, respiratory diseases, respiratory failure, and heart disease than did those in the unvaccinated group, the aORs (95% CI) were 0.87 (0.85–0.90), 0.88 (0.86–0.90), 0.68 (0.64–0.73), and 0.80 (0.78–0.82), respectively. In terms of the hospitalization risks, compared with the unvaccinated group, significant risk reductions (10%–25%) were observed in the vaccinated group (Table 2).

After adjustment, the inpatient expenditure for influenza complications was significantly lower for the vaccinated group than for the unvaccinated group. Reductions in expenditures for influenza and pneumonia, respiratory diseases, respiratory failure, and heart disease were 13%, 13%, 7%, and 8%, respectively (Table 3).

**Table 2:** Comparison of outcome risks between influenza vaccinated and unvaccinated elderly patients with two cohorts.

Variables	Breast Cancer Cohort				General Women Cohort			
	Without Influenza Vaccine (N = 3982)	With Influenza Vaccine (N = 1991)	With Influenza Vaccine vs. without Influenza Vaccine (Ref.)		Without Influenza Vaccine (N = 585,327)	With Influenza Vaccine (N = 585,327)	With Influenza Vaccine vs. without Influenza Vaccine (Ref.)	
	Incident (‰)	Incident (‰)	aOR ^1^	95% CI	Incident (‰)	Incident (‰)	aOR ^2^	95% CI
**All-cause mortality**	17.58	21.09	1.39	0.90–2.16	28.65	16.80	0.58 ***	0.56–0.59
**Emergency admission**								
Influenza and pneumonia	16.57	16.07	1.04	0.65–1.68	22.89	19.93	0.87 ***	0.85–0.90
Respiratory diseases	36.92	30.14	1.02	0.73–1.43	38.88	34.23	0.88 ***	0.86–0.90
Respiratory failure	1.26	2.01	1.61	0.35–7.36	3.81	2.57	0.68 ***	0.64–0.73
Heart disease	16.32	14.57	1.11	0.68–1.82	23.93	19.05	0.80 ***	0.78–0.82
**Hospitalization**								
Influenza and pneumonia	20.59	22.60	1.25	0.83–1.89	31.56	28.34	0.90 ***	0.88–0.92
Respiratory diseases	34.66	41.19	1.38 *	1.01–1.89	46.13	40.54	0.88 ***	0.86–0.90
Respiratory failure	7.53	6.53	0.92	0.44–1.91	12.81	9.55	0.75 ***	0.73–0.78
Heart disease	37.67	44.70	1.27	0.94–1.72	39.48	34.31	0.87 ***	0.85–0.89

Abbreviations: aOR: adjusted odds ratio; CI: confidence interval. * *p* < 0.05; *** *p* < 0.001. All models were analyzed using the generalized estimating equation. ^1^ Extraneous factors adjusted in the model were age, salary, urbanization, CCI, cancer stage, radiotherapy, chemotherapy, hormone treatment, targeted therapy, and health care utilization in the past year (number of outpatient visits, hospitalization, and influenza vaccination status). ^2^ Extraneous factors adjusted in the model were age, salary, urbanization, CCI, and health care utilization in the past year (number of outpatient visits, hospitalization, and influenza vaccination status).

**Table 3:** Comparison of inpatient expenditure between vaccinated and unvaccinated elderly patients with two cohorts.

Variables	Breast Cancer Cohort					General Women Cohort				
	N	Mean	SD	Exp (β) ^1^	95% CI	N	Mean	SD	Exp (β) ^2^	95% CI
Influenza and pneumonia										
Without influenza vaccine (ref.)	82	86,777	104,359			18,470	119,784	184,895		
With influenza vaccine	45	79,516	120,849	0.99	0.65–1.52	16,589	105,472	166,555	0.87 ***	0.85–0.89
Respiratory diseases										
Without influenza vaccine (ref.)	138	136,069	207,251			26,999	141,979	238,490		
With influenza vaccine	82	127,108	317,341	0.89	0.62–1.27	23,730	123,530	218,378	0.87 ***	0.85–0.88
Respiratory failure										
Without influenza vaccine (ref.)	30	247,260	212,202			7500	258,238	341,586		
With influenza vaccine	13	223,307	197,064	0.81	0.48–1.34	5591	240,661	335,844	0.93 ***	0.90–0.97
Heart disease										
Without influenza vaccine (ref.)	150	121,610	181,776			23,111	117,837	180,032		
With influenza vaccine	89	142,199	230,031	1.10	0.81–1.50	20,083	107,633	168,938	0.92 ***	0.90–0.93

Abbreviations: ref., reference group; CI, confidence interval; and SD, standard deviation. *** *p* < 0.001. All models were analyzed using the generalized estimating equation. ^1^ Extraneous factors adjusted in the model were age, salary, urbanization, CCI, cancer stage, radiotherapy, chemotherapy, hormone treatment, targeted therapy, and health care utilization in the past year (number of outpatient visits, hospitalization, and influenza vaccination status). ^2^ Extraneous factors adjusted in the model were age, salary, urbanization, CCI, and health care utilization in the past year (number of outpatient visits, hospitalization, and influenza vaccination status).

## 4. Discussion

The risk of infection acquisition and the inability to prevent infection through immunization are directly related to the patient’s net state of immunosuppression or disease severity [32]. The greater the immunosuppression degree, the less likely the patient is to respond to immunization [2,3]. Although certain existing vaccines provide some benefit to immunocompromised patients, a vaccine response cannot be assumed [2,3,4,5]. Patients with cancer are at an increased risk of serious infection, although the degree of risk varies based on the underlying malignancy and the immunosuppressive treatment type [2,3,6,7,16,17,18,19,20,21,22].

Many of these infections are vaccine preventable. Patients with cancer are at a risk of infection on the basis of debility, malnutrition, and, in some cases, anatomic obstruction (e.g., lung masses obstructing bronchial drainage) [2,3,33]. Vaccines are crucial for patients with cancer; however, ideally, they should not be administered during immunosuppression from chemotherapy immunotherapy because, at such times, they may not be effective, and live vaccines may result in vaccine-derived infections [2,3].

According to the recommendations and limited reports of the influenza vaccine, cancer patients are generally considered eligible for influenza vaccination [2,4,5]. However, not only is the clinical efficacy of influenza vaccines uncertain in patients with cancer receiving chemotherapy and other immunomodulatory agents but also the potential benefit from vaccination is unclear. Until now, it was unclear whether vaccination had a protective effect on clinical outcomes of patients with breast cancer; therefore, the evaluation of the protective effects of influenza vaccines on patients with breast cancer was crucial.

Our study enrolled the largest cohort of elderly patients with breast cancer vaccinated and unvaccinated for influenza to investigate the protective effects of influenza vaccine in patients with breast cancer by using the head-to-head PSM method, which allows the design of an observational (nonrandomized) study and mimics some characteristics of an RCT [23]. The PSM method can decrease the selection bias between influenza vaccinated and unvaccinated patients with breast cancer [23]. All potential confounding factors associated with our endpoints were matched using the PSM method and showed no statistically significant differences between two groups (Table 1).

The advantages of the PSM design in our study was that difficulties and ethical problems with the recruitment of elderly patients with breast cancer into the vaccinated and unvaccinated groups in an RCT could be avoided. An RCT to evaluate the protective effect of influenza vaccine in elderly patients with breast cancer is almost impossible due to the old age and ethical problems. Therefore, the head-to-head PSM design is the optimal for examining the protective effects of influenza vaccine on clinical outcomes in elderly patients with breast cancer.

Until now, data to assess the protective effects of influenza vaccination on clinical outcomes, such as all-cause death, emergency admission, or hospitalization due to influenza-related complications in vaccinated and unvaccinated patients with breast cancer patients were lacking. Most studies had indirect data regarding the antibody response after influenza vaccination [2,3,6,7,16,17,18,19,20,21,22]. However, patients with breast cancer might receive vaccination after breast cancer diagnosis, and the antibody response might be different after breast cancer treatment [6,7].

Although some studies have suggested influenza vaccination administration approximately 2–4 weeks before breast cancer treatment [6,7], no real-world data existed regarding influenza-related complications between vaccinated and unvaccinated elderly patients with breast cancer. Our study is the first to examine the clinical outcomes of influenza vaccine in elderly patients with newly diagnosed breast cancer. No significant protective effect of clinical outcomes was observed (Table 2 and Table 3). The rates of emergency admission or hospitalization due to pneumonia, respiratory disease, respiratory failure, and heart diseases for elderly patients with breast cancer receiving influenza vaccination were high compared with those unvaccinated; however, the findings did not reach significance.

On the other hand, we performed a subgroup analysis of protective effects and different adjuvant treatments for breast cancer, such as chemotherapy, radiotherapy, target therapy, or hormone therapy (Figure 1). However, after adjustment of all cofounding factors (Table 1), no significant differences were observed between vaccinated and unvaccinated elderly patients with breast cancer, irrespective of adjuvant treatments. Some studies have shown that the antibody response might be suppressed after chemotherapy or radiotherapy, which did not contribute to a protective immune response to active vaccination [2,3]; however, some studies have shown contrasting conclusions [4,5].

Thus, no solid evidence exists to suggest the protective effect of influenza vaccination in patients with cancer receiving cancer treatments based on the aforementioned studies with analysis conducted using the antibody response [2,3,4,5,6,7,16,17,18,19,20,21,22]. Our real-world clinical findings for protective effects of influenza vaccine were established based on clinical outcomes instead of the antibody response (Table 2 and Table 3) and did not find evidence supporting the protective effects of influenza vaccination in elderly patients with breast cancer, regardless of any adjuvant treatments (Figure 1). The possible reason for ineffective protective effects to active vaccination is mental stress-related impaired immunity [34,35,36].

To clarify the protective effects of influenza vaccination on other populations, we enrolled the general elderly women as a positive control cohort to assess influenza vaccine validity [37]. The influenza vaccine validity was proven by the protective effects of the vaccination on all clinical outcomes and medical care consumption for influenza complications in the general elderly women who were vaccinated compared with those who were unvaccinated [37]. Our positive control proved and verified that the vaccine could not elicit a protective immune response in elderly patients with breast cancer but could elicit a protective immune response in the general elderly women (Table 2 and Table 3).

The reasons for the ineffective immune response to the influenza vaccine in elderly patients with breast cancer might be multifactorial, including chemotherapy, radiotherapy, and mental stress [2,3,34,35,36]. The protective effect of influenza vaccination in relatively younger populations has been reported for the reduction of ischemic stroke risk, hemorrhage stoke risk, and dementia risk in Taiwan [24,38,39]. However, these are no data to examine the protective effect of influenza vaccination in young breast cancer patients.

This study aimed to examine the protective effect of influenza vaccination in elderly breast cancer patients, and the younger individuals were out of the scope of this study. The younger patients with breast cancer receiving influenza vaccination are also an important public health issue. In the future, it will be necessary to conduct specialized research to examine the protective effect of influenza vaccination on the young population in Taiwan.

The strength of our study is that this is the leading study comparing the effects of influenza vaccination in two population-based cohorts, elderly patients with breast cancer and general elderly women. Moreover, this is the first and largest study to demonstrate that influenza vaccine might have no association with protective effects on the clinical outcomes and medical care consumption of elderly patients with breast cancer in comparison with those who are unvaccinated for influenza.

Therefore, our clinical outcomes will be valuable for future public health policy establishment and shared decision making for influenza vaccine use in elderly patients with newly diagnosed breast cancer. According to our findings, regular influenza vaccine administration for elderly patients with newly diagnosed breast cancer may be reconsidered with certain contraindications for vaccination. On the other hand, implementing the vaccination of close contacts of patients with breast cancer may be a more important strategy for enhancing protection of those fragile patients.

This study has certain limitations. First, because all participants of this study were Asians, the extrapolation of our findings to non-Asian populations is not suitable.

However, influenza infection in Asians may be clinically similar to that in Caucasians [40]. Furthermore, future research goals regarding the disease may be similar in the two populations. Second, the prevalence of male breast cancer was only 1% in the breast cancer population in Taiwan [8]; thus, male patients were excluded from this study. Our hypothesis is the protective effects of influenza vaccination in elderly patients with breast cancer. Ignoring the low proportion (male breast cancer patients) could not overturn the conclusion in the current study based on the law of large numbers. Therefore, we excluded the male breast cancer patients to avoid the bias of different immunity and mortality after vaccinated and cancer treatments.

Third, the diagnoses of all comorbid conditions were based on ICD-9-CM or ICD-10-CM codes. To verify the diagnostic accuracy, the Health and Welfare Data Center, under the Ministry of Health and Welfare Administration, randomly reviews medical records and interviews patients to ensure that hospitals with outlier chargers or practices are audited and subsequently heavily penalized if malpractice or discrepancies are identified. In addition, the quality and precision of ICD-9-CM codes in Taiwan have been verified and proven by previous studies [41,42]. Finally, although informative, the NHIRD lacks essential information, such as dietary habits or body mass index, which may be risk factors for influenza infection–related mortality.

## 5. Conclusions

Influenza vaccination appears to be effective in reducing all-cause mortality, influenza-related emergency admissions, and hospitalizations when compared to unvaccinated among elderly women in Taiwan. However, for elderly women with newly diagnosed breast cancer, we did not find a significant protective effect of influenza vaccination against the outcome indicators. More studies focusing on identifying strategies to improve the real-world effectiveness of influenza vaccination on the immunocompromised are needed.

## Figures and Tables

**Table 1:** The baseline characteristics of two cohorts after matching.

Variables	Breast Cancer Cohort					General Women Cohort				
	1:2 Matching					1:1 Matching				
	Without		With		*p*-Value	Without		With		*p*-Value
	Influenza Vaccine		Influenza Vaccine			Influenza Vaccine		Influenza Vaccine		
	N	%	N	%		N	%	N	%	
Total	3982	66.67	1991	33.33		585,327	50	585,327	50	
Age (year) ^1^ (mean ± SD)	72.29 ± 5.96		72.42 ± 5.65		0.996	74.89 ± 7.74		74.61 ± 7.25		0.654
65–69	1571	39.45	783	39.33		185,230	31.65	185,381	31.67	
70–74	1119	28.1	561	28.18		127,390	21.76	126,979	21.69	
≥75	1292	32.45	647	32.5		272,707	46.59	272,967	46.63	
Salary (NTD) ^1^					0.738					0.37
≤20,008	1578	39.63	777	39.03		183,417	31.34	183,635	31.37	
20,009–22,800	1302	32.7	641	32.19		239,694	40.95	240,282	41.05	
22,801–38,200	397	9.97	216	10.85		66,595	11.38	66,413	11.35	
≥38,201	705	17.7	357	17.93		95,621	16.34	94,997	16.23	
Urbanization ^1^					0.891					0.715
Level 1	1190	29.88	588	29.53		138,491	23.66	137,900	23.56	
Level 2	1389	34.88	678	34.05		170,650	29.15	170,668	29.16	
Level 3	610	15.32	319	16.02		96,335	16.46	96,361	16.46	
Level 4	513	12.88	249	12.51		97,560	16.67	97,769	16.7	
Level 5	58	1.46	34	1.71		18,750	3.2	18,684	3.19	
Level 6	123	3.09	67	3.37		32,906	5.62	33,278	5.69	
Level 7	99	2.49	56	2.81		30,635	5.23	30,667	5.24	
CCI score ^1,2^					0.85					0.567
0	1065	26.75	537	26.97		155,433	26.55	155,844	26.63	
1	1007	25.29	517	25.97		157,954	26.99	158,133	27.02	
2	481	12.08	245	12.31		106,383	18.17	105,843	18.08	
3	1429	35.89	692	34.76		165,557	28.28	165,507	28.28	
Cancer stage ^1^					0.48					
Stage I	1534	38.52	766	38.47						
Stage II	1869	46.94	913	45.86						
Stage III	579	14.54	312	15.67						
Radiotherapy					0.279					
No	2614	65.65	1335	67.05						
Yes	1368	34.35	656	32.95						
Chemotherapy					<0.001					
No	2305	57.89	1354	68.01						
Yes	1677	42.11	637	31.99						
Target therapy.					0.003					
No	3553	89.23	1825	91.66						
Yes	429	10.77	166	8.34						
Hormone treatment					<0.001					
No	1161	29.16	479	24.06						
Yes	2821	70.84	1512	75.94						

^1^ PSM covariates included in the multivariate logistic regression model. ^2^ Cancer was excluded from CCI score calculation. Abbreviations: NTD: New Taiwan dollars; SD: standard deviation; and CCI: Charlson Comorbidity Index.

## Data Availability

The National Health Insurance Database used to support the findings of this study were provided by the Health and Welfare Data Science Center, Ministry of Health and Welfare (HWDC, MOHW) under license and so cannot be made freely available. Requests for access to these data should be made to HWDC (https://dep.mohw.gov.tw/dos/np-2497-113.html, accessed on 16 July 2022).

## Supplementary Materials

The following supporting information can be downloaded at: https://www.mdpi.com/article/10.3390/vaccines10071144/s1, Figure S1: Flowchart of selection of elderly patients with newly diagnosed breast cancer; Figure S2: Flowchart of selection of general elderly women.
