# Supplementary material for: The Protective Effects of Influenza Vaccination in Elderly Patients with Breast Cancer in Taiwan: A Real-World Evidence-Based Study
Source: Vaccines (Basel). 2022 Jul 19;10(7):1144. doi: 10.3390/vaccines10071144 (PMC9320514; doi:10.3390/vaccines10071144)
Supplement: Supplementary file 1 [file vaccines-10-01144-s001.zip › vaccines-1816473-supplementary.pdf]

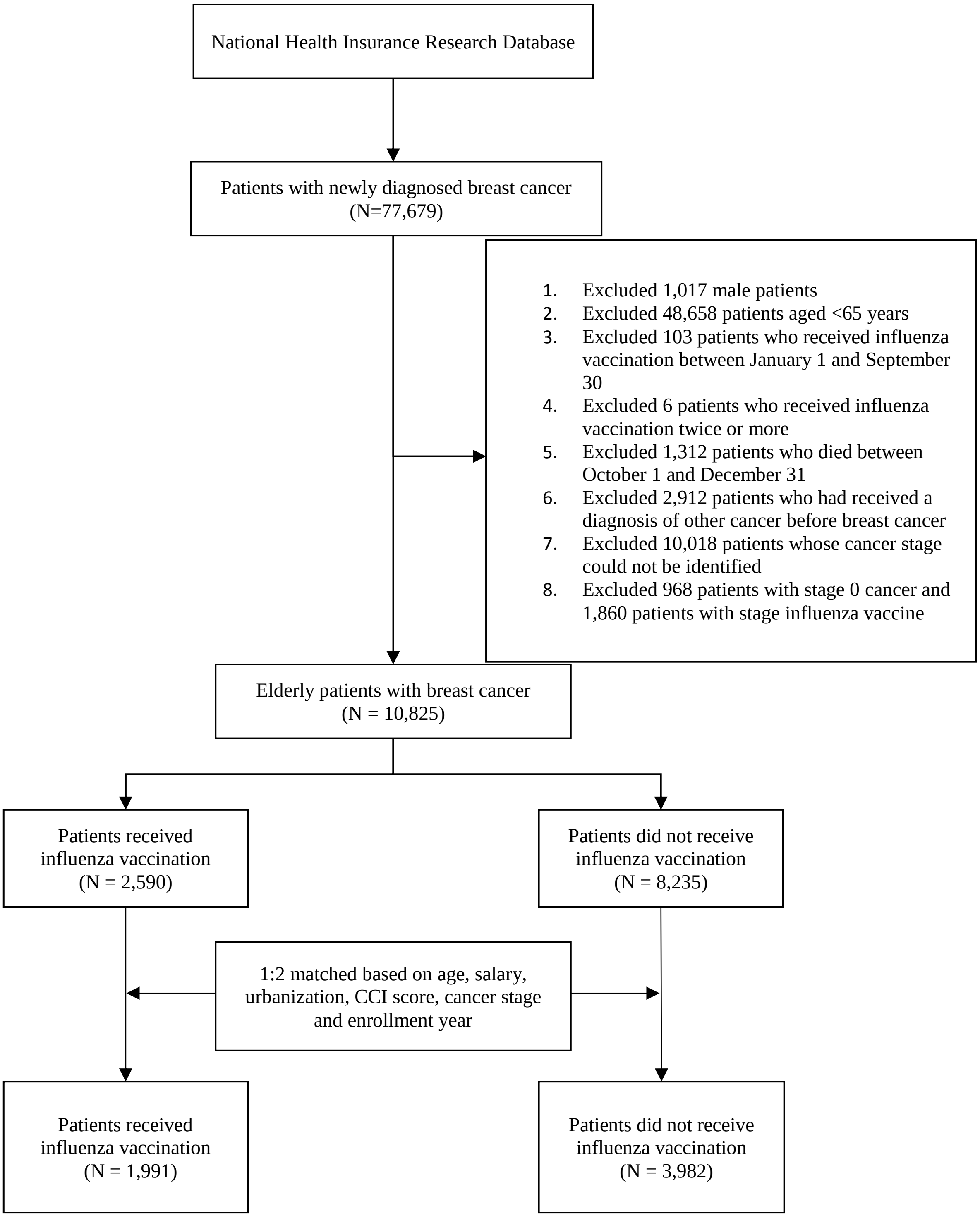

Figure S1. Flowchart of selection of elderly patients with newly diagnosed breast cancer.

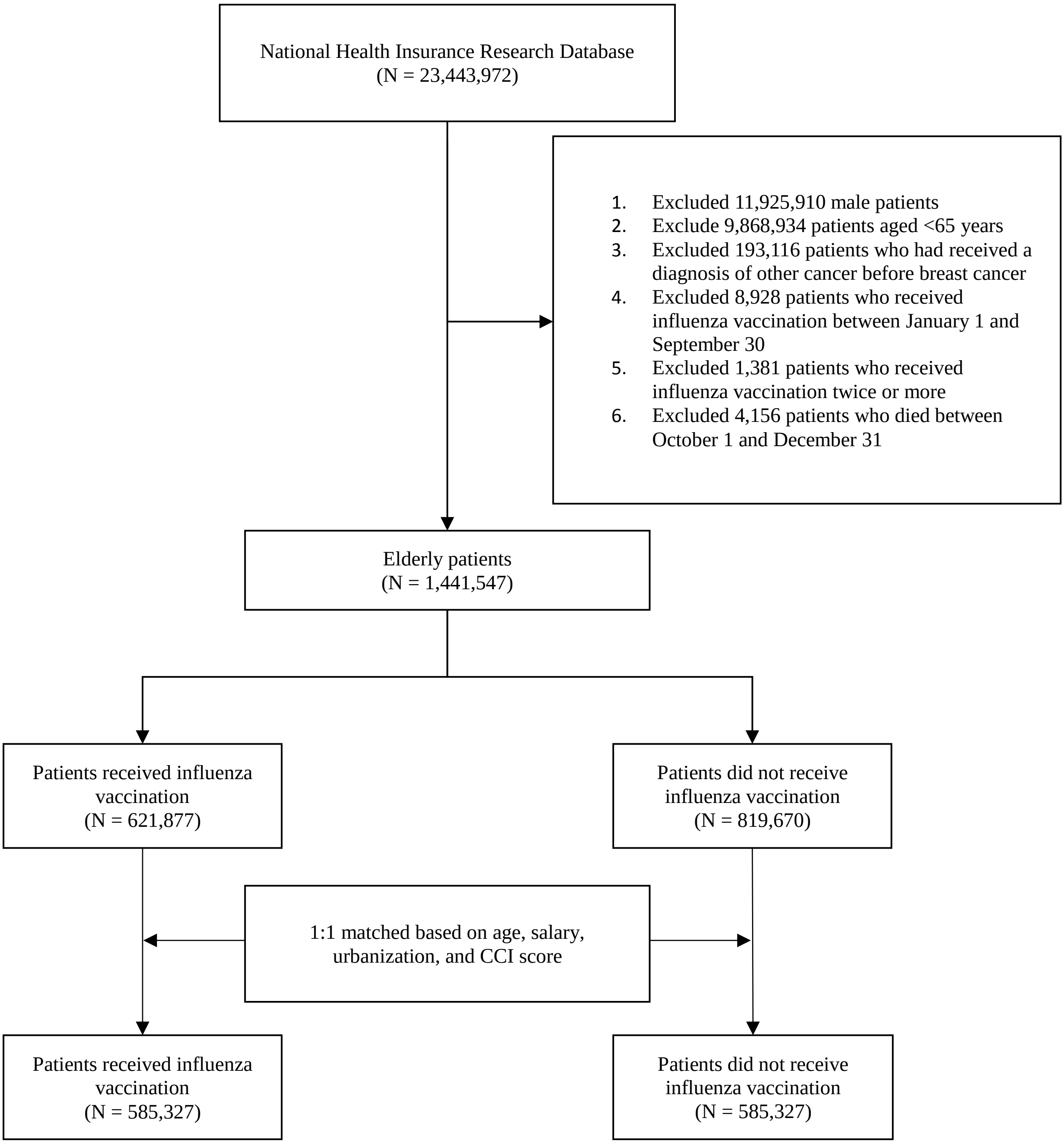

Figure S2. Flowchart of selection of general elderly women
